# ERG is a novel and reliable marker for endothelial cells in central nervous system tumors 

**DOI:** 10.5414/NP300817

**Published:** 2015-04-17

**Authors:** Matthew A. Haber, Amir Iranmahboob, Cheddhi Thomas, Mengling Liu, Amanda Najjar, David Zagzag

**Affiliations:** 1 Microvascular and Molecular Neuro-Oncology Laboratory,; 2 Division of Neuropathology,; 3 Departments of Population Health and Environmental Medicine, and; 4 Department of Neurosurgery, NYU Langone Medical Center, New York, NY, USA

**Keywords:** ERG, immunostain, endothelial cell, CNS tumor, neovascularization

## Abstract

ETS-related gene (ERG) is a transcription factor that has been linked to angiogenesis. Very little research has been done to assess ERG expression in central nervous system (CNS) tumors. We evaluated 57 CNS tumors, including glioblastomas (GBMs) and hemangioblastomas (HBs), as well as two arteriovenous malformations and four samples of normal brain tissue with immunohistochemistry using a specific ERG rabbit monoclonal antibody. In addition, immunostains for CD31, CD34, and α-smooth muscle actin (α-SMA) were performed on all samples. CD31 demonstrated variable and sometimes weak immunoreactivity for endothelial cells. Furthermore, in 1 case of a GBM, CD34 stained not only endothelial cells, but also tumor cells. In contrast, we observed that ERG was only expressed in the nuclei of endothelial cells, for example, in the hyperplastic vascular complexes that comprise the glomeruloid microvascular proliferation seen in GBMs. Conversely, α-SMA immunoreactivity was identified in the abluminal cells of these hyperplastic vessels. Quantitative evaluation with automated methodology and custom Matlab 2008b software was used to calculate percent staining of ERG in each case. We observed significantly higher quantitative expression of ERG in HBs than in other CNS tumors. Our results show that ERG is a novel, reliable, and specific marker for endothelial cells within CNS tumors that can be used to better study the process of neovascularization.

*Both authors have equally contributed to this work. 

## Introduction 

Angiogenesis plays a critical role in various pathologic processes, such as in the pathogenesis of ischemic and neoplastic disorders, including central nervous system (CNS) tumors [1]. For example, in CNS tumors, angiogenesis plays a crucial role in both growth and progression [2]. In addition, the presence or absence of florid microvascular proliferation is an important criterion used in the grading of fibrillary astrocytomas [3] and anti-angiogenesis is one of the therapeutic approaches used in high-grade gliomas [4]. Various CNS tumors, including hemangioblastomas (HBs) and glioblastomas (GBMs), are highly vascularized [1]. In many tumors, hypoxia inducible factor-1α (HIF-1α) is regulated by oxygen concentration and is involved in the activation of many genes, including genes that play a role in survival in anaerobic conditions, as well as angiogenesis [5]. In both HBs and GBMs, the accumulation of HIF-1α leads to increased angiogenesis primarily through the upregulation of vascular endothelial growth factor (VEGF) [1, 6]. For example, in GBMs, the accumulation of HIF-1α protein causes the upregulation of VEGF mRNA in hypoxic pseudopalisading cells adjacent to areas of necrosis [1]. In HBs however, the decreased degradation and subsequent accumulation of HIF-1α protein is caused by a loss of function of the von-Hippel Lindau (VHL) tumor suppressor protein [7], which causes the upregulation of VEGF mRNA in stromal cells [1, 6]. 

ETS-related gene (ERG) is a transcription factor whose expression in normal physiologic conditions is found in endothelial cells and cells of hematopoietic linage [8]. ERG plays a role in endothelial cell migration and has been linked to angiogenesis [9]. For example, a recent study demonstrated that RhoJ, a Rho GTPase family member highly restricted to endothelial cells in several tissues, is a downstream target of ERG and plays a role in capillary morphogenesis, an important step of the angiogenic cascade [10]. ERG also interacts with other transcription factors in order to regulate various genes that are expressed within the endothelial cell lineage, including VE-cadherin, angiopoetin-2, and von Willebrand Factor (vWF) [8]. Moreover, ERG inhibition leads to endothelial cell apoptosis, as well as a decrease in the total number of endothelial cells, endothelial cell-cell connections, and vascularization [11]. 

Much has previously been done to assess ERG expression in endothelial cells within vascular lesions. For instance, one recent study demonstrated strong endothelial immunoreactivity for ERG in both benign and malignant vascular tumors, as well as other vascular lesions, including arteriovenous malformations (AVMs) and papillary endothelial hyperplasia [12]. Furthermore, ERG has previously been shown to be both a sensitive and specific marker for endothelial cells in various vascular malignancies, including angiosarcoma, hemangioma, lymphangioma, Kaposi sarcoma, and hemangioendothelioma [13]. Evidence has also demonstrated the presence of ERG overexpression within various non-vascular neoplasms, including prostate carcinoma, Ewing’s sarcoma, and acute myeloid leukemia [14, 15, 16, 17]. However, a review of the literature indicates that very little has been done to assess the expression of ERG in CNS tumors, or to compare its reliability with that of other endothelial markers, such as CD31 and CD34. Using immunohistochemistry, and a specific rabbit monoclonal antibody, we evaluated ERG expression in CNS tumors. In addition, immunostains for CD31, CD34, and α-smooth muscle actin (α-SMA) were performed on all samples. We also implemented a quantitative analysis of ERG expression throughout different tumor types using a novel computational methodology via a custom Matlab 2008b program. Overall, our results suggest that ERG is a novel, reliable, and specific marker for endothelial cells in CNS tumors that can be used to better study the process of neovascularization. 

## Materials and methods 

### Tissue samples 

This Health Insurance Portability and Accountability Act-compliant study was conducted under a protocol approved by the Institutional Review Board of New York University School of Medicine. We evaluated 57 CNS tumors, which included 16 GBMs, of which 1 case was a recurrent high-grade glioma post-radiation therapy; 4 anaplastic astrocytomas (AAs), 8 HBs, 12 meningiomas, 8 metastatic carcinomas, 2 oligodendrogliomas (OGs), 2 hemangiopericytomas (HPCs), 2 solitary fibrous tumors (SFTs), and 3 schwannomas classified according to the World Health Organization (Table 1); as well as 2 AVMs. The tumors were from 30 female and 27 male patients, with an age range of 19 – 84 years (mean age 53.4). 39 tumors were supratentorial and 18 were infratentorial. Four samples of normal brain tissue removed in the course of surgical exposure were used as controls. When present, normal brain tissue adjacent to the tumor was also used as an internal control. 

### Immunohistochemistry 

Serial sections were stained for hematoxylin and eosin (H & E) and immunostained with a rabbit monoclonal antibody for ERG (clone EPR3864; 0.8 mg/mL). In addition, mouse monoclonal antibodies were also used to stain sections for CD34 (clone QBEnd/10; 23 mg/mL), CD31 (clone JC70; 0.65 mg/mL), and α-SMA (clone IA4; 0.02 mg/mL). Heat-induced epitope retrieval was done by boiling the deparaffinized tissue sections in 10 mmol/L citrate buffer (pH 6.0) in a 1,200 W microwave oven at 90% output for 64 minutes for ERG, 36 minutes for both CD34 and CD31, and 8 minutes for α-SMA. The sections were allowed to cool to room temperature for 30 minutes and subsequently incubated with secondary antibodies at room temperature overnight on a NexES automated immunostainer (Ventana Medical Systems, Tucson, AZ, USA). We used an anti-rabbit biotinylated goat secondary antibody for ERG and one that was anti-mouse for CD34, CD31, and α-SMA. All primary and secondary monoclonal antibodies were purchased prediluted from Ventana Medical Systems. For each antibody, horseradish peroxidase-conjugated strepavidin with 3,3’-diaminobenzidine was used as the chromogen. Nuclei were lightly counterstained with hematoxylin, and slides were dehydrated and mounted with permanent medium. For each immunostain, control procedures included isotype-matched rabbit and mouse monoclonal antibodies. 

### Matlab quantitative analysis of ERG expression 

**Figure 1.**
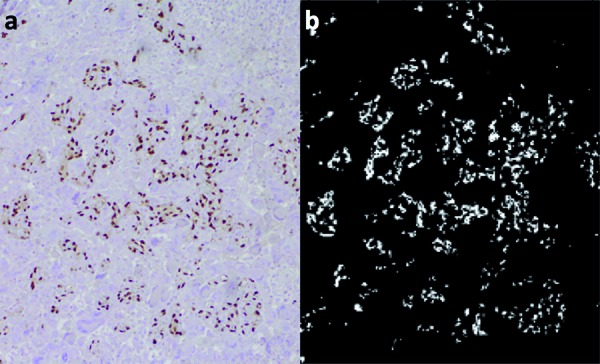
Matlab quantitative determination of EVI. a: Original image of a GBM stained with ERG (100× magnification). b: Demonstration of calculation of degree of ERG staining (EVI = 5.028).

In each sample of tumor and normal brain tissue, the section immunostained for ERG was evaluated using light microscopy at 100× magnification. The two foci containing the most ERG stained capillaries and microvessels, or “hot spots” within each section were located and used for analysis [18]. Computational analysis of ERG expression was performed using a routine spectral clustering threshold method with custom Matlab 2008b software [18], which provided a pixel count quantification of the presence of the immunostain in each section. Pixels were defined as positively ERG-stained with a threshold value of greater than 55%. We defined an ERG vascular index (EVI) as the sum of pixels with ERG-positive nuclear staining divided by the total number of pixels, multiplied by 100. This corresponds to the percent of ERG-positive stained pixels in the image. An example of use of the Matlab methodology for EVI quantification is demonstrated (Figure 1). EVI was calculated for the two foci and the higher value was utilized for quantitative analysis and comparison of different pathologies. The mean EVI for each tumor type and normal brain tissue was calculated and plotted. Statistical analysis with the nonparametric Mann-Whitney test was used to compare percent staining across tumor types and normal brain tissue. 

## Results 

### Immunohistochemical evaluation of gliomas 

**Figure 2.**
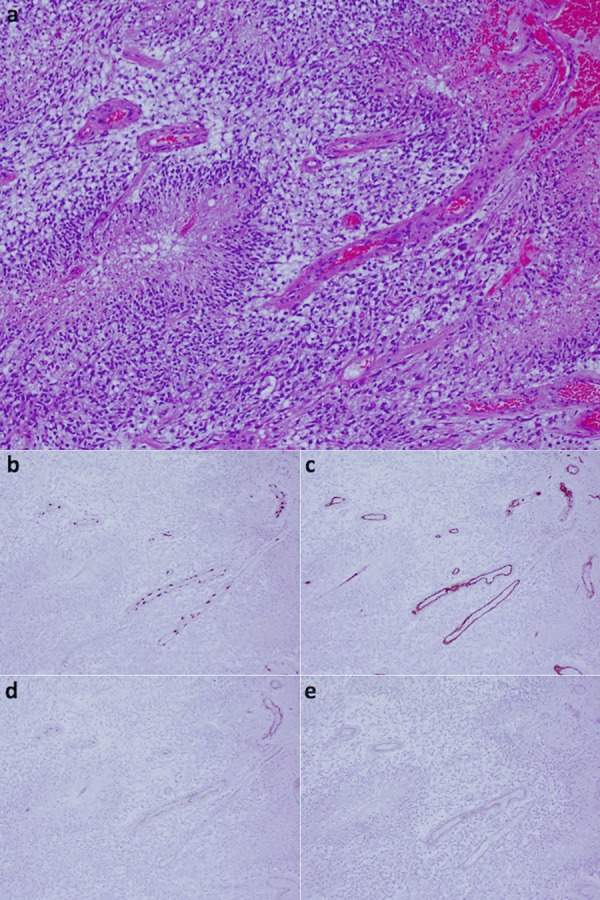
GBM. a: H & E demonstrates pseudopalisading cells surrounding areas of central necrosis, with associated areas of microvascular proliferation. b: ERG exclusively highlights the nuclei of endothelial cells. c: CD34 highlights endothelial cells. d: CD31 weakly highlights endothelial cells. e: α-SMA highlights smooth muscle cells within the walls of vascular channels. The magnification for a: 100×. The magnification for b – e: 50×.

**Figure 3.**
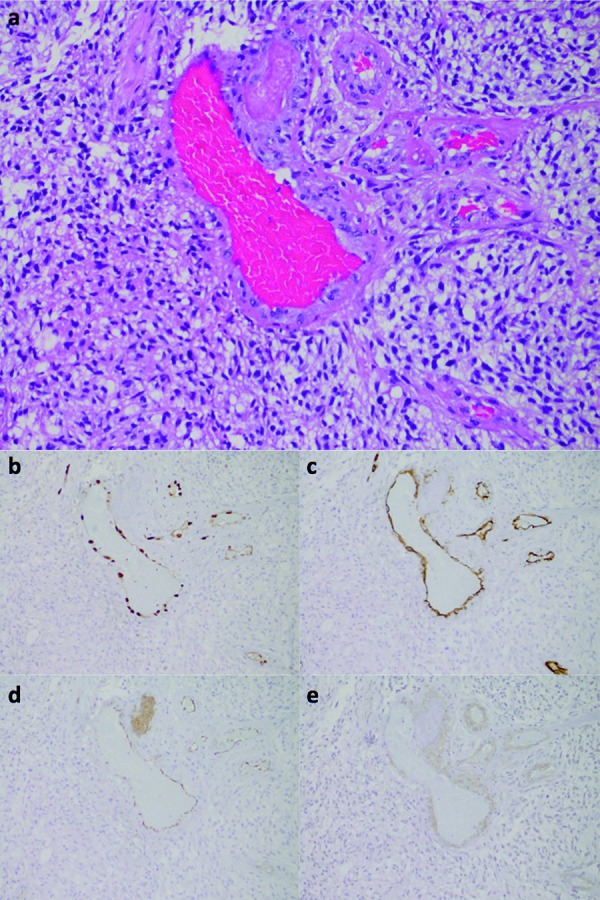
GBM, glomeruloid type. a: H & E demonstrates glomeruloid microvascular proliferation. b: ERG exclusively highlights the nuclei of endothelial cells. c: CD34 highlights endothelial cells. d: CD31 weakly highlights endothelial cells. e: α-SMA highlights smooth muscle cells within the walls of vascular channels. The magnification for a: 100×. The magnification for b – e: 50×.

**Figure 4.**
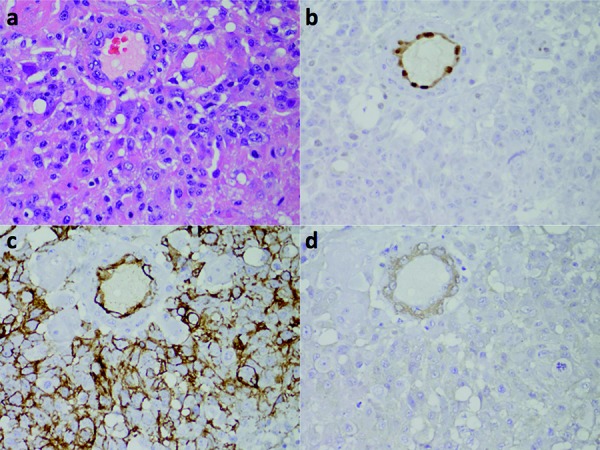
GBM, epithelioid type. a: H & E demonstrates a vascular lumen. b: ERG exclusively highlights the nuclei of endothelial cells. c: CD34 highlights not only endothelial cells, but also tumor cells. d: α-SMA highlights smooth muscle cells within the wall of a vascular channel (a – d: 200× magnification).

In all 15 GBMs, all 4 AAs, and the 2 OGs, we observed strong nuclear immunoreactivity for ERG exclusively in endothelial cells lining vascular lumens (Figure 2b, Figure 3b, Figure 4b). For example, in the glomeruloid microvascular proliferation composed of hyperplastic vascular complexes adjacent to pseudopalisading cells surrounding areas of necrosis, ERG was only detected in endothelial cells (Figure 3b). In contrast, α-SMA immunoreactivity was detected within the abluminal cells of hyperplastic vessels in GBMs (Figure 2e, Figure 3e, Figure 4d). In the 1 GBM case where microvascular proliferation was absent, endothelial cells were also highlighted by the ERG immunoreactivity. In the post-irradiated GBM, secondary microvascular changes were present and with endothelial cells that were strongly reactive for the ERG immunostain. In GBMs, AAs, and OGs immunoreactivity for CD31 and α-SMA was variable and sometimes weak or even absent within non-hyperplastic vascular channels (Figure 2d, e, Figure 3d, e, Figure 4d), while immunoreactivity for CD34 was moderate (Figure 2c, Figure 3c, Figure 4c). In partially sclerosed vessels α-SMA immunoreactivity was reduced, whereas ERG immunoreactivity was present. In addition, in 1 GBM where ERG only stained endothelial cells (Figure 4b), CD34 stained both endothelial and tumor cells (Figure 4c). ERG-positive endothelial cells were seen at the invasive edge of all GBMs as well. 

### Immunohistochemical evaluation of HBs 

**Figure 5.**
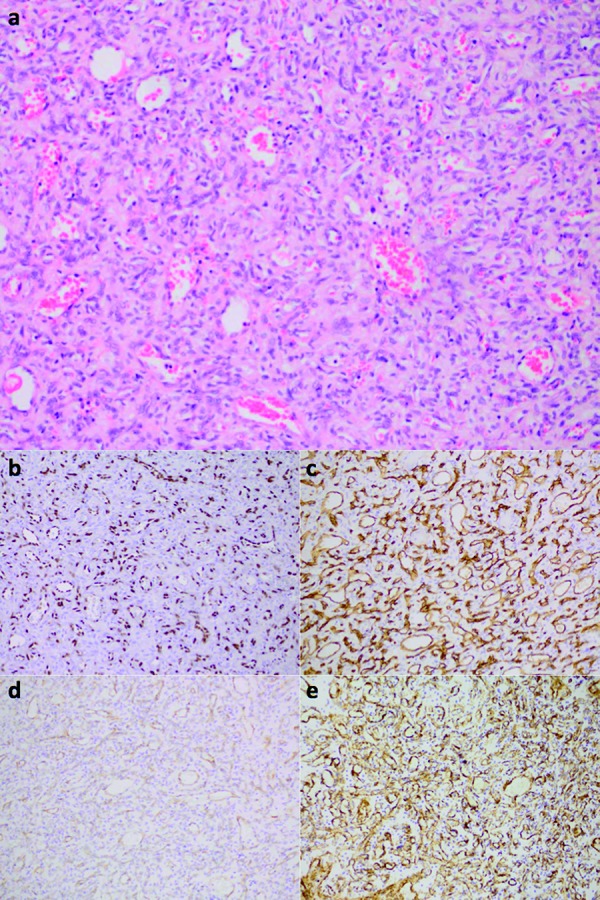
HB. a: H & E demonstrates markedly diffuse microvascular proliferation. b: ERG exclusively highlights the nuclei of endothelial cells. c: CD34 highlights endothelial cells. d: CD31 weakly highlights endothelial cells. e: α-SMA highlights abluminal smooth muscle cells within hyperplastic vascular complexes. The magnification for a: 100×. The magnification for b – e: 50×.

The 8 HBs were highly vascular (Figure 5a). In every case, large areas of tumor showed an anastomosing network of vessels that separated variably abundant groups of stromal cells (Figure 5a). In all 8 HBs, like in GBMs, ERG was only expressed in endothelial cells lining vascular lumens, demonstrating markedly diffuse neovascularization, but was not expressed in stromal cells (Figure 5b). Unlike ERG, CD31 showed variable and sometimes weak immunoreactivity within endothelial cells (Figure 5d), while CD34 showed moderate immunoreactivity (Figure 5c). In contrast to ERG, like in GBMs the α-SMA immunostain highlighted abluminal smooth muscle cells within vessels (Figure 5e). 

### Immunohistochemical evaluation of AVMs, HPCs, meningiomas, metastatic carcinomas, schwannomas, and SFTs 

**Figure 6.**
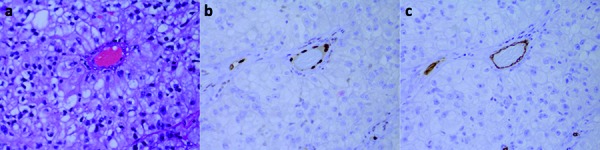
Metastatic carcinoma. a: H & E demonstrates a vascular lumen. b: ERG exclusively highlights the nuclei of endothelial cells. c: CD34 highlights endothelial cells (a – c 200× magnification).

Like in gliomas and HBs, in AVMs, HPCs, meningiomas, metastatic carcinomas (Figure 6a), schwannomas, and SFTs, the nuclei of the endothelial cells lining vascular lumens demonstrated strong immunoreactivity for ERG (Figure 6b). Here again, like in GBMs, AAs, and HBs, endothelial cells were only variably immunoreactive for CD31, and immunoreactivity for CD34 was more intense than for CD31 (Figure 6c). We observed variable α-SMA immunoreactivity within the walls of the vascular channels. 

In the 4 control normal brains, and in cerebral and cerebellar tissue adjacent to 12 GBMs, 3 AAs, and 4 HBs, detectable ERG, CD31, and CD34 immunoreactivity was seen in endothelial cells lining vascular lumens. Here again there was stronger immunoreactivity for ERG as compared to CD31, CD34, and α-SMA. α-SMA immunoreactivity was also observed in the media of arteries and arterioles in the 4 control normal brains, as well as in normal brain distant from 1 GBM and 1 AA. For each tumor case and sample of normal brain tissue used in this study, no staining was observed with isotype-matched rabbit and mouse monoclonal antibody controls in the absence of primary antibody. 

### Matlab quantitative analysis of ERG expression 

**Figure 7.**
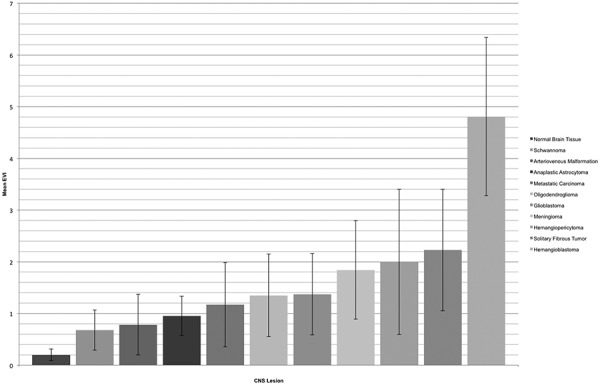
EVI of different CNS lesions, plotted with SD.

The results of the quantitative analysis of ERG immunoreactivity are summarized in Figure 7 and Table 2. We demonstrated significantly more extensive ERG expression in HBs than in other CNS tumors, including GBMs (threshold for statistical significance p < 0.05). Meningiomas and GBMs had the fourth and fifth greatest mean EVIs respectively. As expected, mean EVI was lowest in normal brain tissue. Schwannomas were demonstrated to have the lowest mean EVI of the tumors sampled within our study, and were not found to have significantly more extensive immunostaining for ERG than normal brain tissue. In contrast, meningiomas, metastatic carcinomas, and AAs were found to have significantly more extensive immunostaining for ERG than normal brain tissue. 

## Discussion 

### ERG is a novel, reliable, and specific marker for endothelial cells within CNS tumors 

Our studies demonstrated that in contrast to ERG, CD31 only variably highlighted endothelial cells within CNS tumors and sometimes demonstrated a notably weaker endothelial immunoreactivity. CD31, or platelet endothelial cell adhesion molecule, is a transmembrane glycoprotein expressed in normal physiologic conditions by endothelial cells, platelets, and blood leukocytes, and whose functions include cellular adhesion, platelet activation, and angiogenesis [18, 20]. CD31 is one of the most frequently utilized immunohistochemical markers for endothelial cells, for example, as a marker of angiogenesis in the settings of atherosclerosis and abdominal aortic aneurysm [21], for the quantitative analysis of blood vessels [22], and for determining the degree of neovascularization in a variety of neoplasms, including cervical cancer, ovarian cancer, and Kaposi sarcoma [22, 23, 24]. 

However, in spite of the ubiquitous use of CD31 as a marker for endothelial cells, this immunostain suffers from various shortcomings. For instance, CD31-positive immunostaining has been reported as a less sensitive marker of microvascular density than other markers within neoplasms such as cervical cancer [22]. Furthermore, the expression of CD31 in platelets and blood leukocytes that are adherent to vascular walls may lead to their misidentification as endothelial cells, thus reducing the specificity of this particular immunostain. Additionally, in our study we observed that CD31 only variably and weakly highlighted endothelial cells within CNS tumors (Figure 2d, Figure 3d, Figure 5d), calling into question this immunostain’s use as a marker of such cells. 

CD34 is yet another immunostain with widespread utilization as a marker for endothelial cells. CD34 is a transmembrane glycoprotein expressed in normal physiologic conditions by endothelial cells and hematopoietic stem cells, as well as in dural fibroblastic lesions and non-neoplastic fibrous/leptomeningeal lesions, and whose functions include control of differentiation of stem cells and adhesion [25]. Like CD31, CD34 has been proposed as a sensitive marker for endothelial cells [22], has been used to diagnose vascular tumors [26], and has been used to evaluate the degree of angiogenesis in a variety of neoplasms, including cervical cancer, prostate cancer, and multiple myeloma [22, 27, 28]. 

However, like CD31, CD34 is affected by several drawbacks which should allow us to question the prevalence of its use as an endothelial cell marker. For instance, CD34-positive immunostaining has also been reported in non-vascular cells within CNS tumors, including solitary fibrous tumor and ganglioglioma [29, 30, 31], thus limiting the use of CD34 as a specific marker for endothelial cells. In addition, in 1 case of a GBM in our study, CD34 highlighted not only endothelial cells, but also tumor cells (Figure 4c). 

In our study we have demonstrated that unlike CD31 and CD34, ERG is exclusively expressed in endothelial cells within CNS tumors, lending support to the notion that ERG is a more specific marker for such cells. Furthermore, ERG dependably and intensely highlighted endothelial cells in CNS tumors (Figure 2b, Figure 3b, Figure 4b, Figure 5b, Figure 6b), providing solid evidence that ERG is a more robust endothelial marker than CD31 and CD34 are. In line with these observations and given the various limitations of the CD31 and CD34 immunostains, we recommend that ERG should be used in the future as the primary endothelial immunostain for CNS tumors. 

### Quantitative expression of ERG in endothelial cells in CNS tumors 

Our results revealed significantly higher ERG expression in HBs than in other CNS tumors, including GBMs, which had the fifth greatest mean EVI. These results are consistent with the diffuse, increased vascular density seen in HBs [1], which contrasts with the multifocal and patchy microvascular proliferation in GBMs, for example, adjacent to areas of necrosis [1]. Therefore, although HBs and GBMs are both highly vascularized, the differences in their mean EVI values may be explained by variations in the overall respective homogenous and heterogeneous distribution and landscape of neovascularization within such tumors. The findings that GBMs had a higher mean EVI than AAs and that both GBMs and AAs had a higher mean EVI than normal brain tissue are consistent with the microvascular proliferation seen within high grade gliomas and compatible with the grade assigned to these neoplasms, for the presence or absence of florid microvascular proliferation is an important criterion used in the grading of gliomas [3]. Similarly, meningiomas and metastatic carcinomas of the brain, in contrast to schwannomas, were found to have a significantly higher mean EVI than normal brain tissue. Our results regarding meningiomas and metastatic carcinomas are in line with the important role that angiogenesis plays in such neoplasms [32, 33], providing further evidence that mean EVI correlates with endothelial cell number within CNS tumors. As benign nerve sheath tumors, schwannomas are less likely to have marked angiogenesis than malignant peripheral nerve sheath tumors, also compatible with our results [34]. 

### Use of ERG in understanding the process of neovascularization in gliomas 

In our study we observed that ERG was only expressed in the nuclei of endothelial cells lining vascular lumens in normal brain tissue and within CNS tumors, for example, in the glomeruloid microvascular proliferation seen in GBMs. In contrast, α-SMA immunoreactivity was identified in abluminal cells within the hyperplastic vascular complexes of GBMs [35]. Clearly, the accurate delineation of the cellular components taking part in the microvascular proliferation seen in GBMs is important in order to better understand angiogenesis in CNS tumors. One unresolved and still debated issue related to the cellular components contributing to hyperplastic vessels within GBMs continues to exist. Some have shown that only endothelial cells without the involvement of smooth muscle cells are involved in the microvascular proliferation seen in GBMs [35]. In contrast, other studies have provided experimental data indicating that both endothelial and smooth muscle cells are involved in the microvascular proliferation leading to vascular hyperplasia within glial neoplasms [36]. Our results, which demonstrate the presence of both ERG and α-SMA immunostained cells within vascular lumens, provide novel support for the latter hypothesis of a mixed dual cellular component involved in the glomeruloid microvascular proliferation seen in GBMs, consisting of both endothelial and smooth muscle cells. 

## Conclusion 

In conclusion, we have shown that ERG is a novel and more reliable marker for endothelial cells within CNS tumors than CD31 and CD34 are, adding another tool to the arsenal for the evaluation of CNS tumors. Furthermore, we have demonstrated that ERG expression is significantly higher in HBs than in other types of CNS tumors, including GBMs. Our results help to elucidate the cellular component of the microvascular proliferation of GBMs, furthering our understanding of the development of angiogenesis in CNS tumors. Future studies involving the ERG immunostain may be undertaken in order to better define the biological mechanisms that underlie the process of neovascularization in CNS tumors. 

## Acknowledgments 

This work was supported by grants to DZ from the National Institutes of Health (R01CA100426-01A1, 1R21NS074055-01) and the Musella Foundation. 

## Conflict of interest 

None of the authors reports a conflict of interest. 

**Table 1.** CNS lesions used for ERG immunohistochemistry.

Age	Sex	Location	Pathology
23	F	Left cerebellum	Anaplastic astrocytoma
33	F	Left temporal lobe	Anaplastic astrocytoma
62	M	Left temporal lobe	Anaplastic astrocytoma
62	M	Left frontal lobe	Anaplastic astrocytoma
41	M	Left frontal lobe	Arteriovenous malformation
69	F	Posterior fossa	Arteriovenous malformation
24	F	Right frontal lobe	Glioblastoma
39	M	Right temporal lobe	Glioblastoma
41	F	Right parietal lobe	Glioblastoma
47	M	Left parietal lobe	Glioblastoma
49	M	Right temporal lobe	Glioblastoma
56	M	Right frontal lobe	Glioblastoma
60	M	Left occipital lobe	Glioblastoma (post-irradiated)
60	M	Left occipital lobe	Glioblastoma
60	M	Right occipital lobe	Glioblastoma
61	F	Left parietal lobe	Glioblastoma
61	F	Left temporal lobe	Glioblastoma
64	M	Right temporal lobe	Glioblastoma
67	M	Left frontal lobe	Glioblastoma
67	F	Left frontal lobe	Glioblastoma
68	F	Right temporal lobe	Glioblastoma
71	M	Right frontal lobe	Glioblastoma
19	M	Midline cerebellum	Hemangioblastoma
29	F	Left cerebellum	Hemangioblastoma
33	M	Midline cerebellum	Hemangioblastoma
39	F	Midline cerebellum	Hemangioblastoma
46	F	Left cerebellum	Hemangioblastoma
50	F	Left cerebellum	Hemangioblastoma
50	F	Right cerebellum	Hemangioblastoma
63	M	Right cerebellum	hemangioblastoma
30	F	Right cerebello-pontine angle	Hemangiopericytoma
38	F	Clivus	Hemangiopericytoma
40	F	Left sphenoid wing	Meningioma
43	F	Left frontal lobe	Meningioma
46	M	Right temporo-parietal region	Meningioma
48	F	Right temporal lobe	Meningioma
49	M	Left suboccpital region	Meningioma
55	M	Left frontal lobe	Meningioma
65	F	Right subfrontal region	Meningioma
66	M	Falcine region	Meningioma
69	F	Right sphenoid wing	Meningioma
71	M	Left fronto-parietal region	Meningioma
72	F	Tuberculum sella	Meningioma
73	F	Right retrosigmoid region	Meningioma
54	F	Right cerebellum	Metastatic carcinoma
57	F	Right cerebellum	Metastatic carcinoma
57	F	Left cerebellum	Metastatic carcinoma
57	M	Right parietal lobe	Metastatic carcinoma
61	F	Left cerebellum	Metastatic carcinoma
63	M	Right frontal lobe	Metastatic carcinoma
65	M	Right frontal lobe	Metastatic carcinoma
66	M	Left cerebellum	Metastatic carcinoma
30	M	Right frontal lobe	Oligodendroglioma
56	M	Left frontal lobe	Oligodendroglioma
48	M	Left cerebello-pontine angle	Schwannoma
50	F	Right cerebello-pontine angle	Schwannoma
56	F	Right cerebello-pontine angle	Schwannoma
71	F	Left suboccpital region	Solitary fibrous tumor
84	F	Right suboccipital region	Solitary fibrous tumor
20	M	Right frontal lobe	Normal brain tissue
63	M	Right frontal lobe	Normal brain tissue
63	M	Right frontal lobe	Normal brain tissue
68	F	Left parietal lobe	Normal brain tissue

**Table 2.** Quantitative and statistical analysis of EVI.

Pathology	EVI	SD	N
Hemangioblastoma	4.807	1.528	8
Solitary fibrous tumor	2.228	1.176	2
Hemangiopericytoma	1.998	1.405	2
Meningioma	1.842	0.951	12
Glioblastoma	1.374	0.788	16
Oligodendroglioma	1.35	0.797	2
Metastatic carcinoma	1.17	0.817	8
Anaplastic astrocytoma	0.955	0.378	4
Arteriovenous malformation	0.784	0.588	2
Schwannoma	0.678	0.387	3
Normal brain tissue	0.199	0.114	4

Paired tumors with p-values	Hemangioblastoma	Solitary fibrous tumor	Hemangiopericytoma	Meningioma	Glioblastoma	Oligodendroglioma	Metastatic carcinoma	Anaplastic astrocytoma	Arteriovenous malformation	Schwannoma
Solitary fibrous tumor	0.0889									
Hemangiopericytoma	0.0444	0.667								
Meningioma	0.0001	0.55	0.923							
Glioblastoma	< 0.005	0.209	0.261	0.1						
Oligodendroglioma	0.0444	0.667	0.133	0.55	0.941					
Metastatic carcinoma	0.0002	0.267	0.4	0.135	0.49	0.711				
Anaplastic astrocytoma	0.004	0.133	0.533	0.133	0.494	0.533	0.808			
Arteriovenous malformation	0.0444	0.333	0.667	0.1978	0.471	0.667	0.533	0.533		
Schwannoma	0.0121	0.2	0.4	0.03	0.211	0.4	0.497	0.4	0.8	
Normal brain tissue	0.004	0.133	0.133	0.001	0.0004	0.133	0.0162	0.0286	0.133	0.114

## References

[1] Zagzag D Zhong H Scalzitti JM Laughner E Simons JW Semenza GL Expression of hypoxia-inducible factor 1alpha in brain tumors: association with angiogenesis, invasion, and progression. Cancer. 2000; 88: 2606–2618. 10861440

[2] Hardee ME Zagzag D Mechanisms of glioma-associated neovascularization. Am J Pathol. 2012; 181: 1126–1141. 22858156 10.1016/j.ajpath.2012.06.030 PMC3463636

[3] Louis DN Ohgaki H Wiestler OD Cavenee WK Burger PC Jouvet A Scheithauer BW Kleihues P The 2007 WHO classification of tumours of the central nervous system. Acta Neuropathol. 2007; 114: 97–109. 17618441 10.1007/s00401-007-0243-4 PMC1929165

[4] Plate KH Scholz A Dumont DJ Tumor angiogenesis and anti-angiogenic therapy in malignant gliomas revisited. Acta Neuropathol. 2012; 124: 763–775. 23143192 10.1007/s00401-012-1066-5 PMC3508273

[5] Semenza GL Targeting HIF-1 for cancer therapy. Nat Rev Cancer. 2003; 3: 721–732. 13130303 10.1038/nrc1187

[6] Wizigmann-Voos S Breier G Risau W Plate KH Up-regulation of vascular endothelial growth factor and its receptors in von Hippel-Lindau disease-associated and sporadic hemangioblastomas. Cancer Res. 1995; 55: 1358–1364. 7533661

[7] Vortmeyer AO Falke EA Gläsker S Li J Oldfield EH Nervous system involvement in von Hippel-Lindau disease: pathology and mechanisms. Acta Neuropathol. 2013; 125: 333–350. 23400300 10.1007/s00401-013-1091-z

[8] Nikolova-Krstevski V Yuan L Le Bras A Vijayaraj P Kondo M Gebauer I Bhasin M Carman CV Oettgen P ERG is required for the differentiation of embryonic stem cells along the endothelial lineage. BMC Dev Biol. 2009; 9: 72. 20030844 10.1186/1471-213X-9-72 PMC2803788

[9] Birdsey GM Dryden NH Shah AV Hannah R Hall MD Haskard DO Parsons M Mason JC Zvelebil M Gottgens B Ridley AJ Randi AM The transcription factor Erg regulates expression of histone deacetylase 6 and multiple pathways involved in endothelial cell migration and angiogenesis. Blood. 2012; 119: 894–903. 22117042 10.1182/blood-2011-04-350025

[10] Yuan L Sacharidou A Stratman AN Le Bras A Zwiers PJ Spokes K Bhasin M Shih SC Nagy JA Molema G Aird WC Davis GE Oettgen P RhoJ is an endothelial cell-restricted Rho GTPase that mediates vascular morphogenesis and is regulated by the transcription factor ERG. Blood. 2011; 118: 1145–1153. 21628409 10.1182/blood-2010-10-315275 PMC3148162

[11] Birdsey GM Dryden NH Amsellem V Gebhardt F Sahnan K Haskard DO Dejana E Mason JC Randi AM Transcription factor Erg regulates angiogenesis and endothelial apoptosis through VE-cadherin. Blood. 2008; 111: 3498–3506. 18195090 10.1182/blood-2007-08-105346 PMC2275018

[12] Yaskiv O Rubin BP He H Falzarano S Magi-Galluzzi C Zhou M ERG protein expression in human tumors detected with a rabbit monoclonal antibody. Am J Clin Pathol. 2012; 138: 803–810. 23161713 10.1309/AJCP3K5VUFALZTKC

[13] Miettinen M Wang ZF Paetau A Tan SH Dobi A Srivastava S Sesterhenn I ERG transcription factor as an immunohistochemical marker for vascular endothelial tumors and prostatic carcinoma. Am J Surg Pathol. 2011; 35: 432–441. 21317715 10.1097/PAS.0b013e318206b67b PMC6880747

[14] Demichelis F Fall K Perner S Andrén O Schmidt F Setlur SR Hoshida Y Mosquera JM Pawitan Y Lee C Adami HO Mucci LA Kantoff PW Andersson SO Chinnaiyan AM Johansson JE Rubin MA TMPRSS2:ERG gene fusion associated with lethal prostate cancer in a watchful waiting cohort. Oncogene. 2007; 26: 4596–4599. 17237811 10.1038/sj.onc.1210237

[15] Pigazzi M Masetti R Martinolli F Manara E Beghin A Rondelli R Locatelli F Fagioli F Pession A Basso G Presence of high-ERG expression is an independent unfavorable prognostic marker in MLL-rearranged childhood myeloid leukemia. Blood. 2012; 119: 1086–1087, author reply 1087-1088.. 22282493 10.1182/blood-2011-10-385815

[16] Sashida G Bazzoli E Menendez S Liu Y Nimer SD The oncogenic role of the ETS transcription factors MEF and ERG. Cell Cycle. 2010; 9: 3457–3459. 20814243 10.4161/cc.9.17.13000 PMC3230474

[17] Tsuzuki S Taguchi O Seto M Promotion and maintenance of leukemia by ERG. Blood. 2011; 117: 3858–3868. 21321361 10.1182/blood-2010-11-320515

[18] Weidner N Chapter 14. Measuring intratumoral microvessel density. Methods Enzymol. 2008; 444: 305–323. 19007671 10.1016/S0076-6879(08)02814-0

[19] Shi J Malik J Normalized cuts and image segmentation. IEEE Trans Pattern Anal Mach Intell. 2000; 22: 888–905.

[20] Liu L Shi GP CD31: beyond a marker for endothelial cells. Cardiovasc Res. 2012; 94: 3–5. 22379038 10.1093/cvr/cvs108

[21] DeLisser HM Christofidou-Solomidou M Strieter RM Burdick MD Robinson CS Wexler RS Kerr JS Garlanda C Merwin JR Madri JA Albelda SM Involvement of endothelial PECAM-1/CD31 in angiogenesis. Am J Pathol. 1997; 151: 671–677. 9284815 PMC1857836

[22] Vieira SC Silva BB Pinto GA Vassallo J Moraes NG Santana JO Santos LG Carvasan GA Zeferino LC CD34 as a marker for evaluating angiogenesis in cervical cancer. Pathol Res Pract. 2005; 201: 313–318. 15991838 10.1016/j.prp.2005.01.010

[23] Goodheart MJ Vasef MA Sood AK Davis CS Buller RE Ovarian cancer p53 mutation is associated with tumor microvessel density. Gynecol Oncol. 2002; 86: 85–90. 12079305 10.1006/gyno.2002.6730

[24] Russell Jones R Orchard G Zelger B Wilson Jones E Immunostaining for CD31 and CD34 in Kaposi sarcoma. J Clin Pathol. 1995; 48: 1011–1016. 8543622 10.1136/jcp.48.11.1011 PMC503005

[25] Galloway M CD34 expression in glioblastoma and giant cell glioblastoma. Clin Neuropathol. 2010; 29: 89–93. 20175958 10.5414/npp29089

[26] Miettinen M Lindenmayer AE Chaubal A Endothelial cell markers CD31, CD34, and BNH9 antibody to H- and Y-antigens--evaluation of their specificity and sensitivity in the diagnosis of vascular tumors and comparison with von Willebrand factor. Mod Pathol. 1994; 7: 82–90. 7512718

[27] Bettencourt MC Bauer JJ Sesterhenn IA Connelly RR Moul JW CD34 immunohistochemical assessment of angiogenesis as a prognostic marker for prostate cancer recurrence after radical prostatectomy. J Urol. 1998; 160: 459–465. 9679898

[28] Pruneri G Ponzoni M Ferreri AJ Decarli N Tresoldi M Raggi F Baldessari C Freschi M Baldini L Goldaniga M Neri A Carboni N Bertolini F Viale G Microvessel density, a surrogate marker of angiogenesis, is significantly related to survival in multiple myeloma patients. Br J Haematol. 2002; 118: 817–820. 12181051 10.1046/j.1365-2141.2002.03654.x

[29] Sawada N Ishiwata T Naito Z Maeda S Sugisaki Y Asano G Immunohistochemical localization of endothelial cell markers in solitary fibrous tumor. Pathol Int. 2002; 52: 769–776. 12588446 10.1046/j.1440-1827.2002.t01-1-01423.x

[30] Bisceglia M Galliani C Giannatempo G Lauriola W Bianco M D’angelo V Pizzolitto S Vita G Pasquinelli G Magro G Dor DB Solitary fibrous tumor of the central nervous system: a 15-year literature survey of 220 cases (August 1996-July 2011). Adv Anat Pathol. 2011; 18: 356–392. 21841406 10.1097/PAP.0b013e318229c004

[31] Blümcke I Wiestler OD Gangliogliomas: an intriguing tumor entity associated with focal epilepsies. J Neuropathol Exp Neurol. 2002; 61: 575–584. 12125736 10.1093/jnen/61.7.575

[32] Fidler IJ The role of the organ microenvironment in brain metastasis. Semin Cancer Biol. 2011; 21: 107–112. 21167939 10.1016/j.semcancer.2010.12.009

[33] Lou E Sumrall AL Turner S Peters KB Desjardins A Vredenburgh JJ McLendon RE Herndon JE McSherry F Norfleet J Friedman HS Reardon DA Bevacizumab therapy for adults with recurrent/progressive meningioma: a retrospective series. J Neurooncol. 2012; 109: 63–70. 22535433 10.1007/s11060-012-0861-0 PMC3404217

[34] Miracco C Montesco MC Santopietro R Spina D d’Amore ES Tosi P Ninfo V Proliferative activity, angiogenesis, and necrosis in peripheral nerve sheath tumors: a quantitative evaluation for prognosis. Mod Pathol. 1996; 9: 1108–1117. 8972469

[35] Rojiani AM Dorovini-Zis K Glomeruloid vascular structures in glioblastoma multiforme: an immunohistochemical and ultrastructural study. J Neurosurg. 1996; 85: 1078–1084. 8929498 10.3171/jns.1996.85.6.1078

[36] Wesseling P Vandersteenhoven JJ Downey BT Ruiter DJ Burger PC Cellular components of microvascular proliferation in human glial and metastatic brain neoplasms. A light microscopic and immunohistochemical study of formalin-fixed, routinely processed material. Acta Neuropathol. 1993; 85: 508–514. 7684179 10.1007/BF00230490

